# Modelling inter-individual variability in acute and adaptive responses to interval training: insights into exercise intensity normalisation

**DOI:** 10.1007/s00421-023-05340-y

**Published:** 2023-11-15

**Authors:** Arthur Henrique Bossi, Ulrike Naumann, Louis Passfield, James Hopker

**Affiliations:** 1 School of Sport and Exercise Sciences, University of Kent, Canterbury, Kent UK; 2 School of Applied Sciences, Edinburgh Napier University, Edinburgh, UK; 3 The Mountain Bike Centre of Scotland, Peel Tower, Glentress, Peebles, UK; 4 IQVIA, Frankfurt, Germany; 5 Faculty of Kinesiology, University of Calgary, Calgary, AB Canada

**Keywords:** Intensity prescription, Relative intensity, Intermittent exercise, Individual response, Non-responder, Trainability

## Abstract

**Purpose:**

To investigate the influence of exercise intensity normalisation on intra- and inter-individual acute and adaptive responses to an interval training programme.

**Methods:**

Nineteen cyclists were split in two groups differing (only) in how exercise intensity was normalised: 80% of the maximal work rate achieved in an incremental test (%$\dot{\text{W}}$_max_) vs. maximal sustainable work rate in a self-paced interval training session (%$\dot{\text{W}}$_max-SP_). Testing duplicates were conducted before and after an initial control phase, during the training intervention, and at the end, enabling the estimation of inter-individual variability in adaptive responses devoid of intra-individual variability.

**Results:**

Due to premature exhaustion, the median training completion rate was 88.8% for the %$\dot{\text{W}}$_max_ group, but 100% for the %$\dot{\text{W}}$_max-SP_ the group. Ratings of perceived exertion and heart rates were not sensitive to how intensity was normalised, manifesting similar inter-individual variability, although intra-individual variability was minimised for the %$\dot{\text{W}}$_max-SP_ group. Amongst six adaptive response variables, there was evidence of individual response for only maximal oxygen uptake (standard deviation: 0.027 L·min^−1^·week^−1^) and self-paced interval training performance (standard deviation: 1.451 W·week^−1^). However, inter-individual variability magnitudes were similar between groups. Average adaptive responses were also similar between groups across all variables.

**Conclusions:**

To normalise completion rates of interval training, %$\dot{\text{W}}$_max-SP_ should be used to prescribe relative intensity. However, the variability in adaptive responses to training may not reflect how exercise intensity is normalised, underlining the complexity of the exercise dose–adaptation relationship. True inter-individual variability in adaptive responses cannot always be identified when intra-individual variability is accounted for.

**Supplementary Information:**

The online version contains supplementary material available at 10.1007/s00421-023-05340-y.

## Introduction

It is commonly believed that the magnitude of physiological adaptations varies between individuals undertaking the same exercise training programme (Bouchard et al. 1999; Vollaard et al. 2009; Coakley and Passfield 2018; Montero and Lundby 2017; Astorino et al. 2018; Bonafiglia et al. 2019; McLellan and Skinner 1981; Preobrazenski et al. 2019; Weatherwax et al. 2019; Del Giudice et al. 2020; Hecksteden et al. 2018b). A major factor behind this phenomenon is suggested to be genetics (Mann et al. 2014; Meyler et al. 2021), estimated to account for approximately 50% of the changes in maximal oxygen uptake ($\dot{\text{V}}$O_2max_) (Bouchard et al. 1999, 2011). However, it has been proposed that methods of exercise intensity normalisation in experimental studies do not provide comparable metabolic stress across participants (Mann et al. 2013; Iannetta et al. 2020; Jamnick et al. 2020; Vollaard et al. 2009; Meyler et al. 2021, 2023), contributing to variability in the extent to which training adaptations occur (Mann et al. 2014; Meyler et al. 2021, 2023). For example, when exercise intensity is normalised as a percentage of maximal heart rate, a method with known limitations (Katch et al. 1978; Mann et al. 2013; Iannetta et al. 2020; Jamnick et al. 2020), $\dot{\text{V}}$O_2max_ changes following identical training interventions separated by a washout are only moderately correlated (*r* = 0.31) (Del Giudice et al. 2020). Refining the scientific basis of exercise intensity prescription is, therefore, crucial to understand adaptive response heterogeneity.

The optimal method for exercise intensity normalisation may vary depending on target population, intensity domain of training (i.e. moderate, heavy, very heavy, or severe; see Rossiter (2011) for review), and exercise pattern (i.e. continuous or intermittent) (Mann et al. 2013; Jamnick et al. 2020; Meyler et al. 2023). Moreover, conflicting evidence exists as to whether certain intensity prescription methods could minimise adaptive variability (Weatherwax et al. 2019; McLellan and Skinner 1981; Preobrazenski et al. 2019). Typically, researchers compare two groups undertaking the same training programme but using different normalisation methods to set individual work rates (Weatherwax et al. 2019; McLellan and Skinner 1981; Preobrazenski et al. 2019). In this respect, Weatherwax et al. (2019) reported a reduced inter-individual variability in $\dot{\text{V}}$O_2max_ adaptive responses when exercise intensity domains were individually accounted for, compared with when they were not. McLellan and Skinner (1981), however, reported no differences. Preobrazenski et al. (2019) showed no differences in the magnitude of inter-individual variability of several adaptive responses, including $\dot{\text{V}}$O_2max_, when prescriptions based on the maximal work rate from an incremental test (%$\dot{\text{W}}$_max_) and the talk test were compared (see Reed and Pipe (2014) for talk test details). Nevertheless, they also found that the mean blood lactate concentration ([La^−^]) of the first training session was positively associated with $\dot{\text{V}}$O_2max_ changes within the %$\dot{\text{W}}$_max_ group (Preobrazenski et al. 2019), providing some evidence that individuals experiencing greater metabolic stress may also express larger adaptive response (and vice versa) (Mann et al. 2014, 2013). These inconsistent findings underscore the need for further research to ascertain the extent to which exercise intensity normalisation affects adaptive response variability, and in which contexts.

Interestingly, Montero and Lundby (2017) have demonstrated that a maximised training dose is essential for enhancing the maximal work rate achieved in an incremental test ($\dot{\text{W}}$_max_) and $\dot{\text{V}}$O_2max_ across all individuals within a study cohort. This suggests that McLellan and Skinner (1981) and Preobrazenski et al. (2019) may not have provided their participants with sufficient training stress, making it difficult to untangle potential between-group differences in adaptive variability (Joyner and Lundby 2018). The only study investigating exercise intensity normalisation that assessed adaptive response heterogeneity of a more intense, interval training intervention, compared results with the available literature as opposed to a comparative group, hampering interpretation of their findings (Astorino et al. 2018). Hence, filling this gap is important to elucidate this issue.

For intensive training, maximal self-paced intervals have been employed as a method of exercise intensity normalisation, both in cross-sectional (Brosnan et al. 2000; Villerius et al. 2008; Nicolò et al. 2014) and longitudinal interventions (Seiler and Sylta 2017; Seiler et al. 2013; Rønnestad et al. 2020). Frequently described as “*how elite athletes train*” (Brosnan et al. 2000; Villerius et al. 2008; Rønnestad et al. 2020; Seiler et al. 2013; Seiler and Sylta 2017; Nicolò et al. 2014), this approach is based on the premise that there exists an individualised maximal sustainable work rate for a given interval training format. Provided that work intervals are performed within the very heavy-intensity domain, this concept has precedents in the hyperbolic relationship between work rate and time to exhaustion (Ferguson et al. 2013; Jones and Vanhatalo 2017; Meyler et al. 2023). Surprisingly, there have been no attempts to assess the effectiveness of this method of intensity normalisation in comparison with other approaches such as %$\dot{\text{W}}$_max_.

In the present study, we investigated inter-individual variability in acute and chronic (i.e. adaptive) responses to a training programme in two groups of cyclists. It was hypothesised that the group in which training intensity was prescribed relative to the maximal sustainable work rate in a self-paced interval training session (%$\dot{\text{W}}$_max-SP_) would exhibit less inter-individual variability in acute exercise responses, leading to less variability in adaptive responses, compared with the group in which training intensity was prescribed as %$\dot{\text{W}}$_max_. We also hypothesised that the $\% \dot{\text{W}}_{{{\text{max}}}}$ group would demonstrate a higher proportion of unfinished training sessions, due to miscalculated work rate targets leading to premature exhaustion, potentially compromising the group’s average adaptive responses.

## Methods

### Ethics approval

The research protocols were submitted to and approved by the Research Ethics Committee at the University of Kent (Prop 18_2018_19), in compliance with the Declaration of Helsinki, except for registration in a database. All participants provided written informed consent prior to participating in this study.

### Participants

Nineteen recreationally trained male cyclists (age: 36 ± 10 years, height: 179 ± 8 cm, body mass: 76.3 ± 8.6 kg, $\dot{\text{V}}$O_2max_: 54 ± 6 ml·kg^−1^·min^−1^) volunteered for this study.

### Study design

Participants were involved for 16 weeks (see Table 1), with weeks designated for testing (4 weeks), control (6 weeks), and training intervention (6 weeks). While distinct methods of exercise intensity prescription were used for each group during the training intervention, testing and control phases consisted of identical requirements for all participants. Testing before and after the control phase served as a control against which to gauge the effects of the training interventions (Voisin et al. 2019). Moreover, the testing phase at week 11 enabled the estimation of inter-individual variability in adaptive responses without the need for repeating the training intervention (Hecksteden et al. 2018b). This experimental design makes it possible to estimate inter-individual variability in adaptive responses devoid of intra-individual variability (Voisin et al. 2019; Hecksteden et al. 2018b).

**Table 1:** Timeline of the study

Week	0	1	2	3	4	5	6	7	8	9	10	11	12	13	14	15
Phase	Testing	Control						Testing	Training intervention (%$\dot{\text{W}}$_max_)			Testing	Training intervention (%$\dot{\text{W}}$_max_)			Testing
									Training intervention (%$\dot{\text{W}}$_max-SP_)				Training intervention (%$\dot{\text{W}}$_max-SP_)			
Laboratory visits	3	N/A						3	2	2	2	3	2	2	2	3

%$\dot{\text{W}}$_max_, group in which training intensity was prescribed relative to the maximal work rate achieved in an incremental test; %$\dot{\text{W}}$_max-SP_, group in which training intensity was prescribed relative to the maximal sustainable work rate in a self-paced interval training session; N/A, not applicable

### Testing phase

At consistent times of the day, participants visited the laboratory thrice, at least 48 h apart. In both the first and second visits, participants completed a lactate accumulation test and an incremental test to exhaustion (i.e. duplicate measures were averaged; see page 1 of supplementary material for reliability estimates). In the third visit, participants performed a self-paced interval training session. They were instructed to refrain from intense exercise before testing and to prepare as for competition. Participants were also requested to standardise meals 24 h prior and to consume their last large meal at least 2 h before arrival. The consumption of caffeine was not allowed in the last 12 h before testing. All tests were performed free from distractions, under similar environmental conditions (16–17°C), with participants being cooled with a fan. Maximal encouragement was always provided to warrant representative performances.

The lactate accumulation test started at 100 W, increasing by 50 W after each fifth minute (or 25 W if [La^−^] was ≥ 2.5 mmol·L^−1^), and terminating when [La^−^] reached ≥ 4 mmol·L^−1^. Blood samples taken from a fingertip (at the last 30 s of each 5-min bout) were immediately analysed for [La^−^]. Power output associated with 4 mmol·L^−1^ [La^−^], sometimes referred to as the onset of blood lactate accumulation (Sjödin and Jacobs 1981), was calculated for each cyclist from the relationship between [La^−^] and power output in the last two stages. Before the start, participants chose their preferred cadence for the entire test (91 ± 4 rev·min^−1^). Both the work rates and cadence of the first lactate accumulation test were held constant throughout the study. Breath-by-breath gas exchanges were monitored throughout the test and subsequently smoothed to 30-s averages. Gross efficiency, measured as the ratio between power output and energy expenditure (Hopker et al. 2009), was calculated at 150 W from the mean gas exchanges in the last 2.5 min of the stage. Energy expenditure was estimated assuming negligible protein oxidation according with the equations of Péronnet and Massicotte (1991). All participants met the criterion of a respiratory exchange ratio ≤ 1.0 in all tests.

After the lactate accumulation test, participants cycled for 10 min at a power output between 50 and 100 W. Subsequently, participants completed an incremental test in which work rate increased continuously at 25 W·min^−1^ until voluntary exhaustion, or participants’ inability to maintain cadence above 70 rev·min^−1^. Breath-by-breath gas exchanges were monitored throughout the test and subsequently smoothed to 15-s averages. $\dot{\text{V}}$O_2max_ was identified as the highest 60-s mean oxygen uptake, and $\dot{\text{W}}$_max_ as the mean power output of the last 60 s. Immediately after the incremental test, a blood sample was taken from a fingertip to establish [La^−^], and peak rating of perceived exertion (RPE) was noted.

The self-paced interval training session consisted of six 4-min work intervals interspersed with 2-min active recovery. Participants started immediately after a 10-min warm-up at power outputs between 100 and 150 W. They were required to produce the highest possible amount of work to establish $\dot{\text{W}}$_max-SP_ (i.e. highest possible mean power output across all six work intervals) and received instructions to pace themselves by keeping power reasonably constant between and within work intervals. Recovery intervals had to be performed at power outputs ≤ 70 W. Heart rate was measured as the last-minute average of each work interval. RPE was noted immediately after each work interval.

### Control phase

During this phase, participants did not attend the laboratory. However, they were required to keep their weekly training duration similar to the last two weeks before joining the study, and to avoid structured interval training.

### Training intervention phase

Due to the relatively small number of participants recruited for this study, the first participant was truly randomised, with subsequent participants allocated to one of the two training interventions to keep groups closely matched with regard to dependent variables; i.e. minimisation approach (Hecksteden et al. 2018a). Participants were blinded to their group assignment and unaware of the methods of intensity normalisation used. Both groups attended the laboratory twice per week, at least 72 h apart, to perform interval training sessions consisting of 4-min work intervals interspersed with 2-min active recovery, at predefined work rates. Six training sessions were performed from weeks 8 to 10, and another six from weeks 12 to 14. While in one training intervention (%$\dot{\text{W}}$_max_), the work intervals were performed at 80%$\dot{\text{W}}$_max_ measured on the first incremental test (i.e. visit one of testing; see Table 1); in the other (%$\dot{\text{W}}$_max-SP_), the work intervals were performed at 100%$\dot{\text{W}}$_max-SP_. Recovery intervals were performed at 20% of the work rate prescribed for the work intervals, irrespective of group allocation; i.e. 0.2·(mean_[80%_$\dot{\text{W}}$_max, 100%_$\dot{\text{W}}$_max-SP]_). Participants of both groups were prescribed six work intervals in each training session, except for weeks 8 and 12, in which five work intervals were prescribed to boost their confidence that sessions could be completed. Despite strong encouragement, voluntary exhaustion or inability to maintain cadence above 70 rev·min^−1^ were utilised as criteria to establish individual completion rates in the event of premature termination. Cadence was recorded as the average of each work interval (or the average of completed duration in case of exhaustion), and heart rate as the last-minute average of each work interval (or the average of completed duration if shorter than one minute). RPE was noted immediately after each work interval or at exhaustion. All interval training sessions commenced with a 15-min warm-up and finish with a 3-min cool-down, at, respectively, 60% and 40% of the work rate prescribed for the work intervals, irrespective of group allocation; i.e. 0.6·($\text{mean}_{[80\%\dot{\text{W}}\text{max},\; 100\% \dot{\text{W}}\text{max}-\text{SP}]}$), and 0.4·($\text{mean}_{[80\%\dot{\text{W}}\text{max},\; 100\% \dot{\text{W}}\text{max}-\text{SP}]}$). As $\dot{\text{V}}$O_2max_ gains have been shown to plateau after 3 weeks of high-intensity training at the same work rates (Hickson et al. 1981), training targets were re-adjusted following the testing phase of week 11, no matter if participants exhibited an increase or a decrease in performance. Participants were instructed to perform their remaining training sessions (i.e. outside the laboratory) at work rates below the power output associated with 4 mmol·L^−1^ [La^−^], and to keep weekly training duration similar to the control phase.

### Training intensity determination

The percentage of each prescription benchmark (i.e. 80%$\dot{\text{W}}$_max_ and 100%$\dot{\text{W}}$_max-SP_) was derived based on pilot work with an independent sample of three male and one female cyclists (age: 26 ± 4 years, height: 176 ± 12 cm, body mass: 72.8 ± 15.0 kg, $\dot{\text{V}}$O_2max_: 55 ± 5 ml·kg^−1^·min^−1^). They performed two incremental tests to exhaustion and two self-paced interval training sessions. The averaged work rates for 80%$\dot{\text{W}}$_max_ and 100%$\dot{\text{W}}$_max-SP_ corresponded to 3.59 ± 0.29 and 3.56 ± 0.41 W·kg^−1^, respectively (*P* = 0.705).

### Equipment

Cyclists used their own bikes mounted on a cycle ergometer (Cyclus 2, RBM Elektronik-Automation, Leipzig, Germany). For the lactate accumulation tests, incremental tests, and predefined interval training sessions, the ergometer was set at power mode (i.e. cadence independent). For the self-paced interval training sessions, the ergometer was set at inclination mode (i.e. 0% gradient; cadence dependent), and participants were required to change gears, as if they were riding outdoors. Heart rate was continuously monitored during all sessions through an ANT + belt transmitter (Cyclus 2, RBM Elektronik-Automation, Leipzig, Germany). Elapsed time, power output, heart rate, and cadence were not concealed from participants.

Breath-by-breath gas exchanges were monitored through a metabolic cart (MetaLyzer 3B, Cortex Biophysik, Leipzig, Germany). Prior to every test, calibration was performed according to the manufacturer’s instructions. [La^−^] was assessed using an automatic analyser (Biosen C-Line, EKF Diagnostics, Penarth, UK). RPE was assessed based on the 6–20 Borg’s scale (Borg 1982). The same trained experimenter conducted all testing and training sessions to minimise procedural variability.

### Data analysis

Data were assessed for normality using Shapiro–Wilk’s test and normal quantile plots. To investigate between-group differences in target work rates for the training sessions, independent samples *t* tests were used. Training intervention completion rates were assessed for between-group difference using a Mann–Whitney test. Training RPE, heart rate, and cadence were investigated via linear mixed models with participant as a random effect, and group, training session, and work interval as fixed effects. To identify evidence of between-group differences in the magnitude of inter- and intra-individual variability, models were fitted with homogeneous and heterogeneous inter- and intra-individual variance structures for group.

To investigate between-group differences in the adaptive response variables (i.e. $\dot{\text{V}}$O_2max_, $\dot{\text{W}}$_max_, power output associated with 4 mmol·L^−1^ [La^−^], gross efficiency, $\dot{\text{W}}$_max-SP_, and body mass) prior to the intervention, independent samples *t* tests were used. Differences in adaptive response variables between testing weeks 0, 7, 11, and 15 were assessed using repeated-measures analysis of variance, with Bonferroni pairwise comparisons used to identify where significant differences existed within the data. Linear mixed models, with participant as a random effect, and group and testing occasion as fixed effects, were used to test for a group effect on adaptive response variables’ change from week 0 while controlling for their absolute baseline scores. To investigate inter-individual variability in adaptive responses to training, piecewise linear mixed models were used with participant and participant-by-intervention week interaction as random effects, and control week and intervention week as fixed effects. The standard error of the participant-by-intervention week interaction was used to calculate confidence intervals associated with individual adaptive responses. Individuals whose confidence intervals overlapped ‘0’ were considered non-responders, whereas those whose confidence intervals did not overlap ‘0’ were considered responders or adverse responders based on a positive or negative response, respectively. Optimal models were selected using likelihood ratio tests. Pearson’s correlation was employed to examine the relationship between modelled adaptive responses.

Data were analysed using Prism 8 (GraphPad, San Diego, USA), with model fitting performed in R 4.0.4 (R Foundation for Statistical Computing, Vienna, Austria). Significance level was set at *P* ≤ 0.05, and confidence level was set at 95%. Results are presented as mean ± SD unless otherwise stated. The reader unfamiliarised with linear mixed models is referred to Brown (2021), Naumova et al. (2001), and Pinheiro and Bates (2020).

## Results

### Training intervention

All participants attended all sessions of the training intervention. Target work rates for training are presented in Table 2. No between-group differences were detected for any of the target work rates, at either the first or second half of the intervention (all *P* ≥ 0.220).

**Table 2:** Target work rates for training (W·kg^−1^)

Group	Training intervention (1st half)				Training intervention (2nd half)			
	Warm-up	Work intervals	Recovery intervals	Cool-down	Warm-up	Work intervals	Recovery intervals	Cool-down
%$\dot{\text{W}}$_max_	2.30 ± 0.21	3.89 ± 0.35	0.77 ± 0.07	1.53 ± 0.14	2.35 ± 0.21	3.97 ± 0.36	0.78 ± 0.07	1.57 ± 0.14
%$\dot{\text{W}}$_max-SP_	2.26 ± 0.17	3.76 ± 0.32	0.75 ± 0.06	1.51 ± 0.11	2.29 ± 0.17	3.77 ± 0.32	0.76 ± 0.06	1.53 ± 0.11

%$\dot{\text{W}}$_max_, group in which training intensity was prescribed relative to the maximal work rate achieved in an incremental test; %$\dot{\text{W}}$_max-SP_, group in which training intensity was prescribed relative to the maximal sustainable work rate in a self-paced interval training session

Rates of training completion are presented in Table 3. Due to premature exhaustion, participants of the %$\dot{\text{W}}$_max_ group did not complete the entire sessions as often as participants of the %$\dot{\text{W}}$_max-SP_ group, resulting in lower overall completion rates.

**Table 3:** Training intervention completion rates (%)

Training session	Participants (%$\dot{\text{W}}$_max_)										Participants (%$\dot{\text{W}}$_max-SP_)								
	1	3	5	7	9	11	13	15	17	19	2	4	6	8	10	12	14	16	18
1	100.0	100.0	100.0	100.0	68.0	100.0	66.0	50.0	100.0	100.0	86.0	100.0	100.0	100.0	100.0	100.0	100.0	100.0	100.0
2	88.0	100.0	100.0	100.0	100.0	100.0	100.0	64.0	100.0	68.0	100.0	100.0	100.0	100.0	100.0	100.0	100.0	100.0	100.0
3	88.3	100.0	76.7	100.0	71.7	100.0	88.3	40.0	55.0	56.7	100.0	100.0	100.0	100.0	100.0	100.0	100.0	100.0	100.0
4	100.0	100.0	71.7	100.0	100.0	100.0	66.7	75.0	100.0	55.0	100.0	100.0	100.0	100.0	100.0	100.0	100.0	100.0	100.0
5	100.0	100.0	100.0	100.0	100.0	100.0	88.3	53.3	100.0	55.0	100.0	100.0	100.0	100.0	100.0	100.0	100.0	100.0	100.0
6	100.0	100.0	66.7	100.0	100.0	100.0	100.0	73.3	100.0	58.3	91.7	100.0	100.0	100.0	100.0	100.0	100.0	100.0	100.0
7	100.0	84.0	80.0	100.0	100.0	100.0	70.0	50.0	100.0	100.0	88.0	100.0	100.0	100.0	100.0	100.0	100.0	100.0	100.0
8	100.0	68.0	86.0	100.0	100.0	100.0	86.0	50.0	100.0	100.0	100.0	100.0	100.0	100.0	100.0	100.0	100.0	100.0	100.0
9	60.0	73.3	73.3	100.0	100.0	100.0	53.3	60.0	100.0	100.0	91.7	71.7	100.0	100.0	100.0	100.0	100.0	100.0	100.0
10	90.0	88.3	53.3	100.0	100.0	85.0	86.7	40.0	100.0	100.0	93.3	100.0	100.0	100.0	70.0	100.0	100.0	100.0	100.0
11	50.0	73.3	90.0	100.0	100.0	68.3	73.3	41.7	100.0	100.0	73.3	100.0	100.0	100.0	100.0	100.0	100.0	100.0	100.0
12	100.0	71.7	88.3	100.0	100.0	53.3	91.7	41.7	100.0	100.0	56.7	71.7	100.0	100.0	100.0	100.0	100.0	100.0	100.0

Overall	89.3	88.2	81.6	100.0	95.1	91.8	80.9	53.2	96.0	82.2	89.9	95.0	100.0	100.0	97.4	100.0	100.0	100.0	100.0
Group median [Q1–Q3]	88.8 [81.4–95.3]										100.0 [96.2–100.0]*								

%$\dot{\text{W}}$_max_, group in which training intensity was prescribed relative to the maximal work rate achieved in an incremental test; %$\dot{\text{W}}$_max-SP_, group in which training intensity was prescribed relative to the maximal sustainable work rate in a self-paced interval training session; Q1, 25th percentile; Q3, 75th percentile.

*Denotes significant difference (*P* = 0.003).

There were no between-group differences for RPE, heart rate, or cadence (see %$\dot{\text{W}}$_max-SP_ estimates on page 2 of supplementary material), and there was no evidence of between-group differences in the magnitude of inter-individual variability for these variables (Table 4). In contrast, there was evidence of lower intra-individual variability in acute training responses for the %$\dot{\text{W}}$_max-SP_ group (Table 4).

**Table 4:** Variability in acute exercise responses (SD)

	Inter-individual variability			Intra-individual variability		
	%$\dot{\text{W}}$_max_	%$\dot{\text{W}}$_max-SP_	P	%$\dot{\text{W}}$_max_	%$\dot{\text{W}}$_max-SP_	P
RPE	0.9	0.7	0.360	0.9	0.8	0.005
Heart rate (beats·min^−1^)	9	8	0.915	4	3	< 0.001
Cadence (rev·min^−1^)	6	5	0.534	5	3	< 0.001

%$\dot{\text{W}}$_max_, group in which training intensity was prescribed relative to the maximal work rate achieved in an incremental test; %$\dot{\text{W}}$_max-SP_, group in which training intensity was prescribed relative to the maximal sustainable work rate in a self-paced interval training session; RPE, ratings of perceived exertion

### Training outcomes

**Fig. 1:**
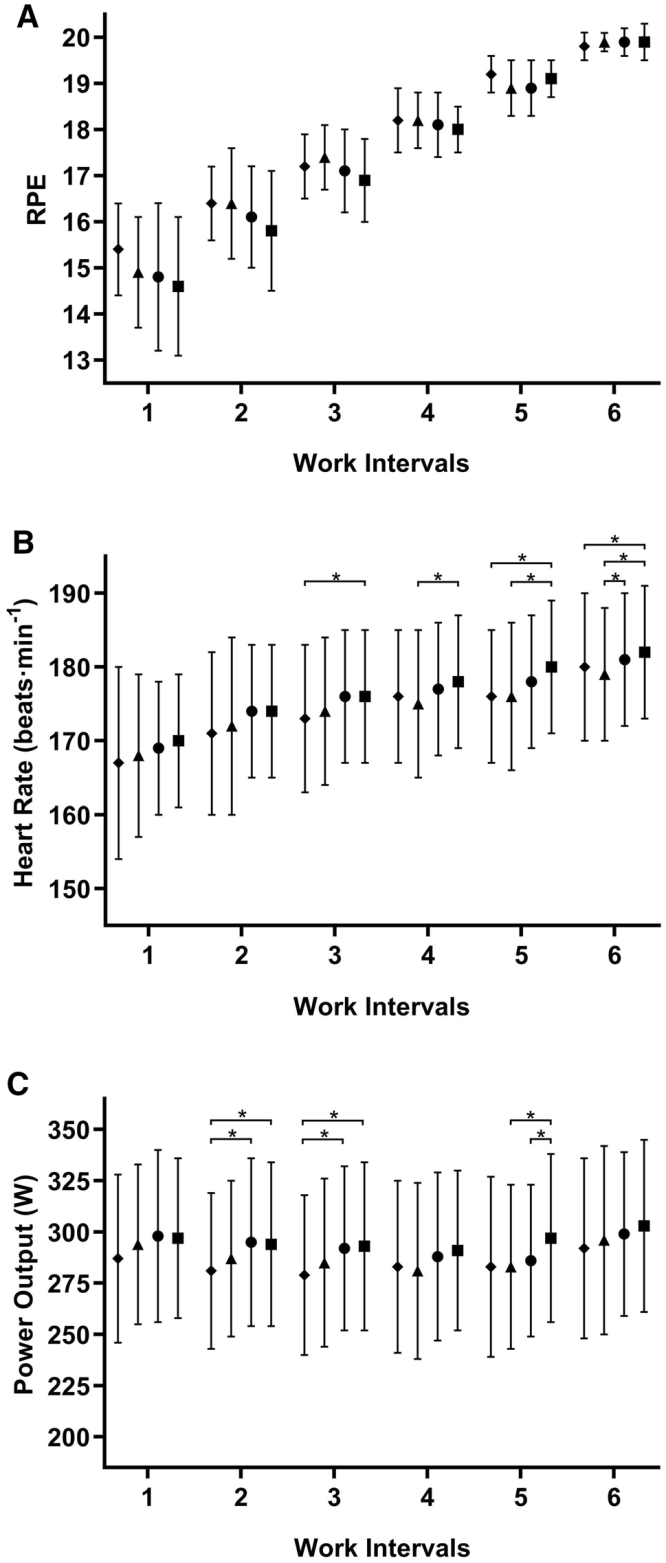
Ratings of perceived exertion (RPE—panel A), heart rate (panel B), and power output (panel C) of each work interval of the self-paced interval training session (mean ± SD). Diamonds, triangles, circles, and squares represent weeks 0, 7, 11, and 15, respectively. * denotes significant difference (all *P* ≤ 0.042)

Participants attended all testing sessions, except for one participant of the %$\dot{\text{W}}$_max-SP_ group that did not attend the second visit of week 0. No between-group differences were detected for any of the adaptive response variables, at either week 0 or week 7 (all *P* ≥ 0.233), suggesting the participant allocation into groups was successful. During the self-paced session, RPEs associated with each work interval were not different between weeks (all *P* ≥ 0.102). This is despite an increased heart rate and power output after the start of the training intervention (see Fig. 1 for details), suggesting that participants consistently adhered to instructions.

**Fig. 2:**
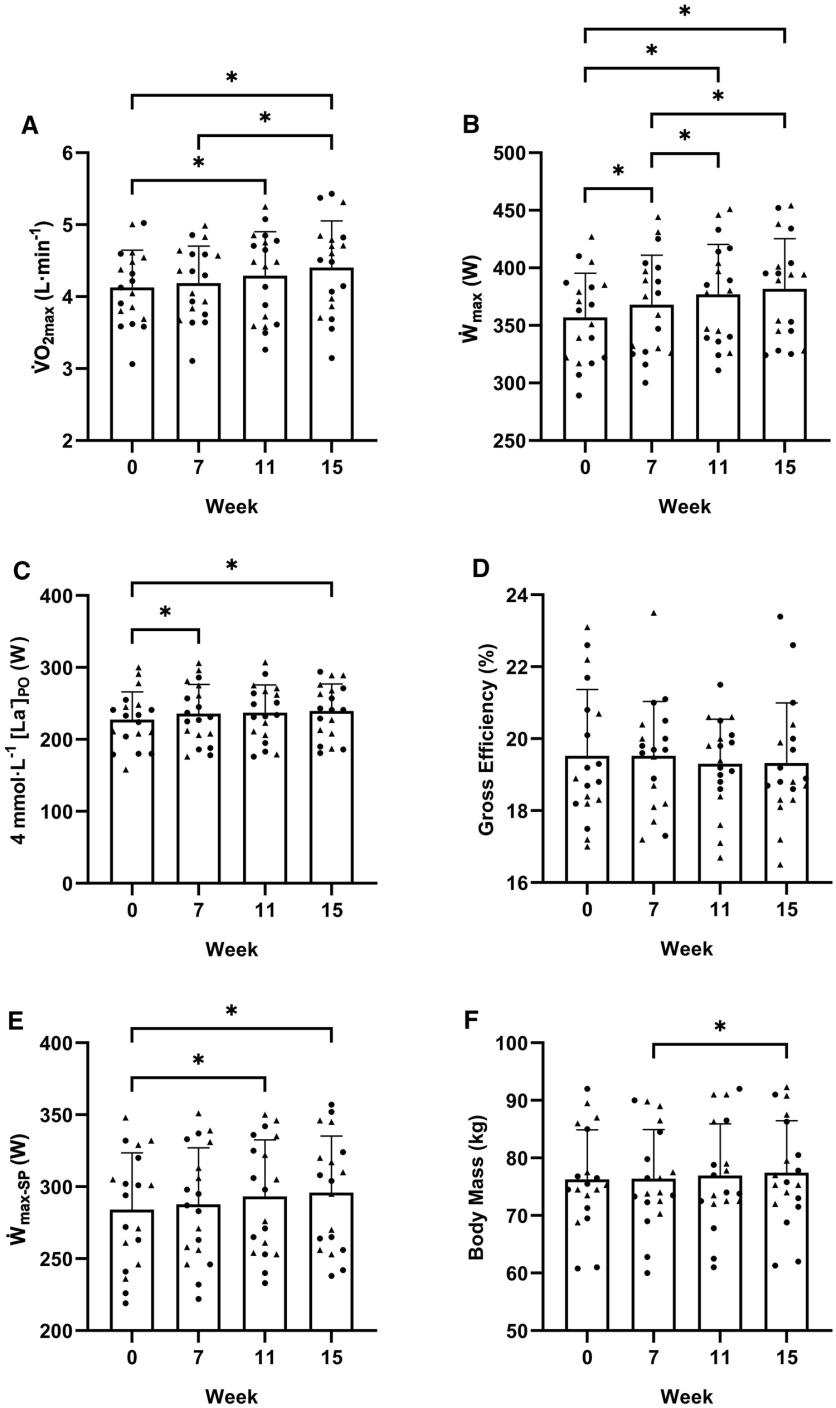
Gross measures (mean ± SD) of maximal oxygen uptake ($\dot{\text{V}}$O_2max_–panel A), maximal work rate in an incremental test ($\dot{\text{W}}$_max_–panel B), power output associated with 4 mmol·L^−1^ blood lactate concentration (4 mmol·L^−1^ [La^−^]_PO_–panel C), gross efficiency (panel D), maximal sustainable work rate in a self-paced interval training session ($\dot{\text{W}}$_max-SP_–panel E), and body mass (panel F). Circles represent individuals of the %$\dot{\text{W}}$_max_ group. Triangles represent individuals of the %$\dot{\text{W}}$_max-SP_ group. See text for group definitions. * denotes significant difference (all *P* ≤ 0.044)

When considering all participants together, changes over the 16 weeks of the study were evident for all adaptive response variables, except gross efficiency (Fig. 2). During the control phase (from week 0 to 7), $\dot{\text{W}}$_max_ and power output associated with 4 mmol·L^−1^ [La^−^] increased by 11 W (*P* < 0.001) and 8 W (*P* = 0.027), respectively, but there was no change for any other adaptive response (all *P* ≥ 0.414). During the training intervention (from week 7 to 15), $\dot{\text{V}}$O_2max_ increased by 0.215 L·min^−1^ (*P* = 0.038), $\dot{\text{W}}$_max_ increased by 14 W (*P* < 0.001), and body mass increased by 1.1 kg (*P* = 0.009). While there was also an increasing trend for $\dot{\text{W}}$_max-SP_ from week 7 to 15 (8 W, *P* = 0.085), it reached statistical significance only compared with week 0 (12 W, *P* = 0.014). Power output associated with 4 mmol·L^−1^ [La^−^] did not increase further from week 7 (*P* = 0.636). When changes in adaptive response variables were modelled, a group difference was evident only for gross efficiency (%$\dot{\text{W}}$_max-SP_ group: − 0.8%, *P* = 0.044). However, adding a testing occasion-group interaction did not further improve the gross efficiency model (*P* = 0.119).

After accounting for intra-individual variability associated with control and intervention phases (see Table 5 for fixed effects), there was evidence of inter-individual variability in adaptive responses for $\dot{\text{V}}$O_2max_ (*P* = 0.003 – see page 3 of supplementary material) and $\dot{\text{W}}$_max-SP_ (*P* = 0.001 – see page 4 of supplementary material). However, adding an intervention week-group interaction as a fixed or random effect did not improve the models (*P* ≥ 0.197 for all model comparisons), indicating that there was no evidence of between-group differences in the magnitude of inter-individual variability for either variable. Accordingly, the confidence intervals for the SD of the individual intervention-week coefficients overlapped substantially ($\dot{\text{V}}$O_2max_: 0.017–0.045 L·min^−1^·week^−1^ for the %$\dot{\text{W}}$_max_ group, and 0.014–0.040 L·min^−1^·week^−1^ for the %$\dot{\text{W}}$_max-SP_ group; $\dot{\text{W}}$_max-SP_: 0.948–2.517 W·week^−1^ for the %$\dot{\text{W}}$_max_ group, and 0.762–2.161 W·week^−1^ for the %$\dot{\text{W}}$_max-SP_ group). Unlike $\dot{\text{V}}$O_2max_ and $\dot{\text{W}}$_max-SP_, there was no evidence of inter-individual variability in adaptive responses for $\dot{\text{W}}$_max_ (*P* = 0.207), power output associated with 4 mmol·L^−1^ [La^−^] (*P* = 0.466), gross efficiency (*P* = 0.348), or body mass (*P* = 0.173).

**Table 5:** Fixed effects upon adaptive responses to training

	$\dot{\text{V}}$O_2max_ (L·min^−1^)	$\dot{\text{W}}$_max_ (W)	4 mmol·L^−1^_PO_ (W)	GE (%)	$\dot{\text{W}}$_max-SP_ (W)	Body mass (kg)
Intercept	4.127	357	227	19.5	284	76.3
Control week	0.009	1.683	1.171	−0.004	0.578	0.015
Intervention week	0.018	0.028	−0.688	−0.023	0.442	0.117

Formula: dependent variable = intercept + control week coefficient · *x*  + intervention week coefficient · *x*. For control week, *x* = 0 to 15; for intervention week, *x* = 0 to 8 (where intervention week 1 corresponds to control week 8). $\dot{\text{V}}$O_2max_, maximal oxygen uptake; $\dot{\text{W}}$_max_, maximal work rate in an incremental test; 4 mmol·L^−1^_PO_, power output associated with 4 mmol·L^−1^ blood lactate concentration; GE, gross efficiency; $\dot{\text{W}}$_max-SP_, maximal sustainable work rate in a self-paced interval training session

**Fig. 3:**
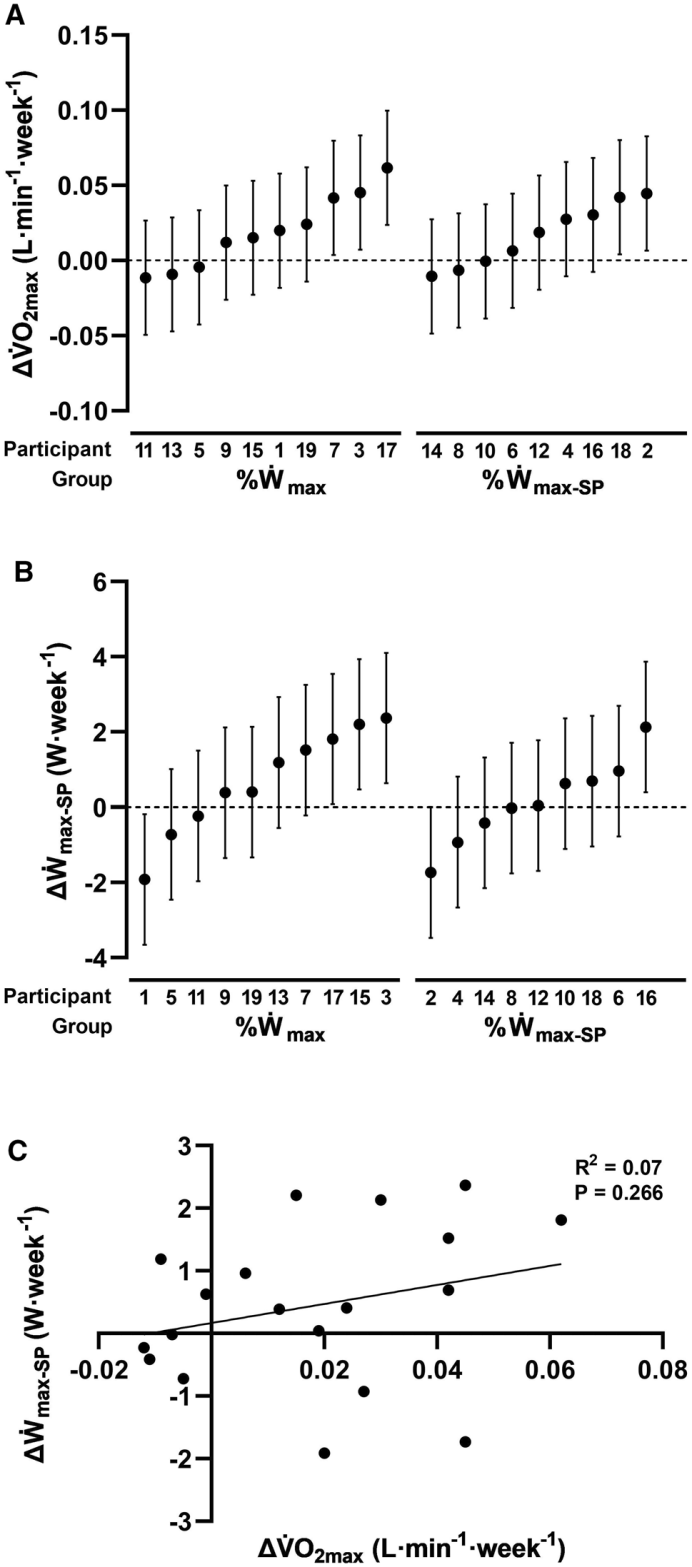
Individual estimates with confidence intervals for weekly changes in maximal oxygen uptake ($\Delta \mathop {\text{V}}\limits^{.}$O_2max_–panel A) and maximal sustainable work rate in a self-paced interval training session (Δ$\dot{\text{W}}$_max-SP_–panel B) beyond the increase associated with the control phase, and related scatterplot (panel C)

Both $\dot{\text{V}}$O_2max_ and $\dot{\text{W}}$_max-SP_ models yielded large residual errors relative to the variability in intervention-week slopes (see pages 3 and 4 of supplementary material), resulting in wide confidence intervals for individual responses (Fig. 3—panels A and B), and making it difficult to categorise most participants. There were three and two responders for $\dot{\text{V}}$O_2max_ in the %$\dot{\text{W}}$_max_ and %$\dot{\text{W}}$_max-SP_ groups, respectively, with the remaining participants being categorised as non-responders. There were three responders, six non-responders, and one adverse responder for $\dot{\text{W}}$_max-SP_ in the %$\dot{\text{W}}$_max_ group; and one responder, seven non-responders, and one adverse responder in the %$\dot{\text{W}}$_max-SP_ group. However, modelled $\dot{\text{V}}$O_2max_ and $\dot{\text{W}}$_max-SP_ responses were not correlated (Fig. 3—panel C).

## Discussion

We investigated acute and chronic (i.e. adaptive) responses to a training programme in which recreationally trained cyclists were split into two groups differing in exercise intensity normalisation methods, but with identical prescriptions otherwise. The main findings are: a) performance in a maximal self-paced interval training session (i.e. %$\dot{\text{W}}$_max-SP_) may be used to normalise the exercise intensity of interval training performed at predefined work rates, particularly if premature exhaustion is to be avoided; b) after accounting for sources of intra-individual variability, there was evidence of adaptive response heterogeneity for $\dot{\text{V}}$O_2max_ and $\dot{\text{W}}$_max-SP_ only, but no between-group differences in magnitude; and c) average adaptive responses were not different between groups, meaning that the higher prevalence of incomplete training sessions in the %$\dot{\text{W}}$_max_ group, due to premature exhaustion, did not compromise participants’ training effect.

### Self-paced performance to normalise interval training intensity

Research on exercise intensity normalisation has been ongoing since the late 1970’s, with Katch et al. (1978) and McLellan and Skinner (1985) amongst the first to challenge the efficacy of percentages of maximal heart rate and $\dot{\text{V}}$O_2max_. Subsequent studies have also identified shortcomings in other traditional methods, namely %$\dot{\text{W}}$_max_ and $\dot{\text{V}}$O_2max_/heart rate reserve (Iannetta et al. 2020; Jamnick et al. 2020; Mann et al. 2013; Marini et al. 2021). Yet, all these methods continue to be used (Vollaard et al. 2009; Bouchard et al. 1999, 2011; Hecksteden et al. 2018b; Coakley and Passfield 2018; Bonafiglia et al. 2019; Montero and Lundby 2017; Del Giudice et al. 2020), most likely due to the limited empirical support for alternative approaches, such as the delta concept, which considers different physiological anchors (Lansley et al. 2011; McLellan and Skinner 1985; Meyler et al. 2023), critical power modelling (Ferguson et al. 2013; Jones and Vanhatalo 2017; Meyler et al. 2023), and maximal self-paced intervals (Villerius et al. 2008; Seiler and Sylta 2017; Nicolò et al. 2014; Brosnan et al. 2000). Accordingly, the present investigation reveals that prescribing exercise intensity of interval training as 100%$\dot{\text{W}}$_max-SP_ minimises performance variability between individuals compared with 80%$\dot{\text{W}}$_max_. Only occasionally (8.3% of the sessions), did participants of the %$\dot{\text{W}}$_max-SP_ group experience premature exhaustion throughout the training intervention, with a median completion rate of 100%. In contrast, premature exhaustion was very common (44.2% of the sessions) amongst participants of the %$\dot{\text{W}}$_max_ group, with a median completion rate of 88.8% (see Table 3 for individual data). This was despite a similar exercise intensity between groups on average (see Table 2). These data thus reinforce previous critiques of %$\dot{\text{W}}$_max_ (Iannetta et al. 2020; Jamnick et al. 2020) and substantiate the use of 100%$\dot{\text{W}}$_max-SP_ for interval training intensity normalisation.

The relationship between work rate and sustainable duration is largely individual, particularly for intermittent exercise (Ferguson et al. 2013; Jones and Vanhatalo 2017; Meyler et al. 2023). Accordingly, it makes sense to establish a common duration, and allow individuals to select the maximal sustainable work rate, instead of presuming that a single variable (e.g. %$\dot{\text{W}}$_max_) is able to predict their exercise capacity. Prescribing exercise at 100%$\dot{\text{W}}$_max-SP_ nevertheless assumes that a) individuals can pace maximal efforts to deliver performances consistent with their capacity; and b) self- and ergometer-paced performances are equivalent when the mean work rate is the same, which may not be universally true (Black et al. 2015; Thomas et al. 2013). Crucially, our data ease concerns about both assumptions. In line with other studies (Villerius et al. 2008; Seiler and Sylta 2017; Nicolò et al. 2014; Brosnan et al. 2000), RPE increased quasi-linearly during the self-paced interval training sessions, approaching 20 in the last work interval (see Fig. 1). Moreover, no differences between testing occasions were detected for the RPEs associated with each work interval, despite an increased heart rate and performance after the start of the training intervention. These observations suggest that performance gains most likely reflected an improved exercise capacity rather than a different approach to the task. As for the self- vs. ergometer-paced performances, only participant 2 consistently struggled to complete training sessions at predefined work rates, with an 89.9% overall completion rate.

While it is tempting to conclude that maximal self-paced intervals should be incorporated into training, replacing intervals at predefined work rates (Rønnestad et al. 2020; Seiler and Sylta 2017; Seiler et al. 2013), some physiological responses such as oxygen uptake are sensitive to large power output variations within (Bossi et al. 2020) and between work intervals (Ferguson et al. 2013). Whether variability in power output distribution would contribute to increased inter-individual variability in acute and adaptive responses to training is unclear. We, therefore, preliminarily recommend that maximal self-paced intervals are used only to determine %$\dot{\text{W}}$_max-SP_.

### RPE and heart rate as indicators of exercise response variability

Even though a lower performance variability between individuals was detected for the %$\dot{\text{W}}$_max-SP_ compared with the %$\dot{\text{W}}$_max_ group, RPE and heart rate data only partially corroborate this finding. Within the %$\dot{\text{W}}$_max-SP_ group, the magnitude of intra-, but not inter-individual variability, was lower for both RPE and heart rate (see Table 4). From an intra-individual perspective, this outcome likely stems from the fact that participants of the %$\dot{\text{W}}$_max-SP_ group consistently completed their training sessions, stopping at the same timepoint, whereas premature exhaustion occurred at different timepoints when participants of the %$\dot{\text{W}}$_max_ group struggled. This is expected, due to normal day-to-day performance variability (Midgley et al. 2007) plus the combined effects of gradual training adaptation and work rate adjustment at week 11 (see RPE on page 2 of supplementary material for evidence of the latter effects). From an inter-individual perspective, our findings align with those of Meyler et al. (2023), who showed that heart rate, oxygen uptake, and [La^−^] do not always reflect between-group differences in inter-individual variability in performance. Therefore, physiological and perceptual responses to high-intensity training may not be as sensitive as performance to quantify variability and inform the normalisation of exercise intensity. Alternatively, an effective normalisation of exercise intensity based on performance may not ensure uniform physiological and perceptual responses across individuals. While more studies are required to elucidate these hypotheses, it is important to underscore that RPE and heart rate data were modelled to factor in the fixed effects of group, training session, and work interval, meaning that our estimates are conservative compared with other studies (Lansley et al. 2011; Scharhag-Rosenberger et al. 2010; Vollaard et al. 2009; Katch et al. 1978; Meyler et al. 2023), and certainly closer to the true inter-individual variability (Voisin et al. 2019; Hecksteden et al. 2018b). Unless the pitfalls inherent to the analysis of raw variability are avoided (Voisin et al. 2019; Hecksteden et al. 2018b; Williamson et al. 2017; Atkinson et al. 2019), future investigations are unlikely to clarify our findings.

### Adaptive response heterogeneity and the impact of intra-individual variability

Since the influential work of Bouchard et al. (1999) investigating the heritability of $\dot{\text{V}}$O_2max_ responses to training, several authors have claimed that the extent to which each individual adapts to a standardised programme is fairly unique (Vollaard et al. 2009; Coakley and Passfield 2018; Bonafiglia et al. 2019; Preobrazenski et al. 2019; Astorino et al. 2018; Weatherwax et al. 2019; Montero and Lundby 2017; Del Giudice et al. 2020; Hecksteden et al. 2018b). However, apart from Hecksteden et al. (2018b), they did not account for all sources of variability affecting the observed inter-individual variability (Voisin et al. 2019; Hecksteden et al. 2018b; Williamson et al. 2017; Atkinson et al. 2019), prompting questions as to the existence of true adaptive response heterogeneity (Williamson et al. 2017). By following the best design and analytical practices (Voisin et al. 2019; Hecksteden et al. 2018b), we demonstrate that variability between individuals in $\dot{\text{W}}$_max_, power output associated with 4 mmol·L^−1^ [La^−^], gross efficiency, and body mass responses to an interval training programme is likely a manifestation of intra-individual variability associated with the control phase and/or the intervention phase itself. This interpretation is strengthened by the use of averaged duplicate measures to minimise day-to-day biological and technical fluctuations, facilitating the identification of a true inter-individual variability (if present) (Voisin et al. 2019). Adaptive response heterogeneity was nevertheless detected for $\dot{\text{V}}$O_2max_ and $\dot{\text{W}}$_max-SP_, even though the latter variable was not analysed in duplicates. Together, these distinct outcomes indicate that inter-individual variability in training adaptations can occur, although it may be difficult to demonstrate statistically when all confounding sources of variability are accounted for and/or the magnitude of changes associated with an intervention is relatively small.

Upon re-analysis of the HERITAGE Family Study data (Bouchard et al. 1999), Shephard et al. (2004) have demonstrated that the true inter-individual variability in $\dot{\text{V}}$O_2max_ adaptive responses was much smaller than originally estimated. The raw SD of 0.010 L·min^−1^·week^−1^ represented in reality 0.007 or 0.006 L·min^−1^·week^−1^, whether a 2-day or a 2-week test–retest coefficient of variation for $\dot{\text{V}}$O_2max_ was considered, respectively, to factor in the intra-individual variability expected for assessments conducted 20 weeks apart. Given that Shephard et al. (2004) were not able to account for intra-individual variability associated with identical training programmes, either through repeated testing or repeated interventions (Voisin et al. 2019; Hecksteden et al. 2018b), the 0.007–0.006 L·min^−1^·week^−1^ figure likely still overestimates the true inter-individual variability. Accordingly, the question that arises is whether recreationally trained cyclists, as employed herein, are more susceptible to adaptive response heterogeneity than sedentary individuals, as employed in the HERITAGE Family Study (Bouchard et al. 1999) and elsewhere (Hecksteden et al. 2018b). The SD for $\dot{\text{V}}$O_2max_ responses reached 0.027 L·min^−1^·week^−1^ in the current study, notably higher than those estimated by Shephard et al. (2004) (see above), and Hecksteden et al. (2018b) as 0.042 ml·kg^−1^·min^−1^·week^−1^. In theory, sedentary individuals have untapped genetic potential for $\dot{\text{V}}$O_2max_ improvements, unlike recreationally trained cyclists, resulting in inconsistent adaptive gains within the latter cohort. Further studies are necessary to test this hypothesis.

Interestingly, we also found evidence of adaptive response heterogeneity for $\dot{\text{W}}$_max-SP_, implying that the extent to which participants improved intermittent self-paced performance varied (with a SD of 1.451 W·week^−1^). While no comparable studies exist, this may suggest that $\dot{\text{W}}$_max-SP_ is characterised by a high signal-to-noise ratio, being sensitive to small changes in exercise capacity, and thus suitable as an intensity prescription benchmark. Conversely, adaptive response heterogeneity was not detected for $\dot{\text{W}}$_max_. These findings are consistent with the fact that 100%$\dot{\text{W}}$_max-SP_ successfully normalised the completion rates of interval training, while 80%$\dot{\text{W}}$_max_ led to premature exhaustion in 44% of the sessions.

### Similar group-level adaptive responses despite contrasting rates of training completion

Despite the evidence in favour of $\dot{\text{W}}$_max-SP_ as a benchmark for interval training prescription, there were no between-group differences in the magnitude of adaptive variability, either for $\dot{\text{V}}$O_2max_ or $\dot{\text{W}}$_max-SP_. Contrary to our hypothesis, adaptive responses may be too complex to reflect the manipulation of a single element of training prescription (i.e. how exercise intensity is normalised). Mann et al. (2014) and Meyler et al. (2021) have listed factors unrelated to the training intervention that are known to affect adaptive responses, including genetics, nutrition, and recovery from one exercise session to another. While genetics is believed to account for approximately 50% of the inter-individual variability in $\dot{\text{V}}$O_2max_ responses to a training programme (Bouchard et al. 1999, 2011), the isolated or combined impact of training prescription, nutrition, and recovery remains unclear. Given that the average changes in $\dot{\text{V}}$O_2max_, %$\dot{\text{W}}$_max_, power output associated with 4 mmol·L^−1^ [La^−^], gross efficiency, $\dot{\text{W}}$_max-SP_, and body mass were also not different between groups, it may be speculated that fine-tuning exercise intensity is irrelevant from an adaptive point of view. To shed light on this possibility, a literature overview is instructive.

McLellan and Skinner (1981) compared the inter-individual variability in $\dot{\text{V}}$O_2max_ responses between groups; one in which exercise intensity was normalised as %$\dot{\text{V}}$O_2max_, and another in which exercise intensity was normalised relative to the first ventilatory threshold (%VT_1_). No between-group differences were detected for the magnitude of inter-individual variability. Likewise, the dataset of Preobrazenski et al. (2019), which included $\dot{\text{V}}$O_2max_, $\dot{\text{W}}$_max_, and power output associated with 4 mmol·L^−1^ [La^−^], displays a similar magnitude of inter-individual variability between groups (i.e. 65%$\dot{\text{W}}$_max_ vs. first negative stage of the talk test). Current results, therefore, corroborate these previous findings. In contrast, by comparing the heart rate reserve method with an individualised approach using the heart rate associated with each ventilatory threshold, Weatherwax et al. (2019) concluded that how exercise intensity is normalised affects the inter-individual variability in $\dot{\text{V}}$O_2max_ responses. However, a detailed inspection reveals that the individualised approach group trained at a higher intensity on average and made a larger $\dot{\text{V}}$O_2max_ gain. As Weatherwax et al. (2019) relied on the responder counting approach, which has been shown to reflect the magnitude of mean differences rather than inter-individual differences (Atkinson et al. 2019), their inference could be questioned.

Even though the evidence mostly indicates that adaptive response heterogeneity is not directly influenced by how exercise intensity is normalised, we cannot discard a small contribution. For example, Preobrazenski et al. (2019) revealed that the mean [La^−^] of the first training session in a series was positively associated with $\dot{\text{V}}$O_2max_ changes within their 65%$\dot{\text{W}}$_max_ group. Similarly, Gaskill et al. (2001) showed that training intensity in the HERITAGE Family Study, originally normalised as %$\dot{\text{V}}$O_2max_ (Bouchard et al. 1999), accounted for 26% of the gains in the oxygen uptake associated with VT_1_ when expressed as %VT_1_. In other words, the higher the intensity relative to VT_1_, the greater the VT_1_ gain (Gaskill et al. 2001). These two studies provide some evidence that the metabolic stress experienced by each individual is associated with their adaptive response (Mann et al. 2014, 2013). Thus, sample sizes of less than twenty participants per group, as employed herein and elsewhere (McLellan and Skinner 1981; Preobrazenski et al. 2019; Weatherwax et al. 2019), may not be enough to investigate adaptive response heterogeneity from an intensity normalisation perspective. This possibility requires careful consideration by those designing future studies.

### Limited utility of the responder counting approach

The responder counting approach has been frequently adopted to investigate inter-individual variability in adaptive responses to a training programme (Bouchard et al. 1999; Vollaard et al. 2009; Coakley and Passfield 2018; Montero and Lundby 2017; Astorino et al. 2018; Bonafiglia et al. 2019; Weatherwax et al. 2019; Del Giudice et al. 2020; Hecksteden et al. 2018b). While definitions for responders, non-responders, and adverse responders vary between studies, constituting a problem in itself (see Hecksteden et al. (2018b) and Voisin et al. (2019) for overview), it has been argued that this approach is flawed for two main reasons: a) observed responses may simply reflect intra-individual variability of different sorts (Voisin et al. 2019; Hecksteden et al. 2018b; Williamson et al. 2017; Atkinson et al. 2019); and b) the number of responders, non-responders, and adverse responders of a sample is expected to conform with a normal distribution, reflecting deviations of the mean, rather than the true magnitude of adaptive response heterogeneity (Atkinson et al. 2019). In light of these criticisms, we used the standard error of the participant-by-intervention week interaction to calculate confidence intervals associated with individual adaptive responses for $\dot{\text{V}}$O_2max_ and $\dot{\text{W}}$_max-SP_. As results demonstrate, most participants were classified as non-responders due to the uncertainty with which individual responses are estimated. For both $\dot{\text{V}}$O_2max_ and $\dot{\text{W}}$_max-SP_ models, there was a large residual error compared with the inter-individual variability in intervention-week slopes, suggesting a great level of intra-individual variability. These findings, therefore, corroborate previous demonstrations that the responder counting approach may be untenable (Atkinson et al. 2019; Hecksteden et al. 2018b).

### Investigating inter-individual variability in performance as a meaningful target

Interestingly, modelled gains in $\dot{\text{V}}$O_2max_ and $\dot{\text{W}}$_max-SP_ resulting from the training intervention did not correlate, despite $\dot{\text{V}}$O_2max_ being generally considered the main endurance performance determinant (Joyner and Coyle 2008). Björklund et al. (2007) also found no correlation between $\dot{\text{V}}$O_2max_ and time to exhaustion during an interval training session, whilst Daniels et al. (1978) and Vollaard et al. (2009) reported no association between changes in $\dot{\text{V}}$O_2max_ and time-trial performance following a training intervention. Together, these findings suggest that, within athletic populations, the scientific interest for inter-individual variability in adaptive responses should perhaps shift from $\dot{\text{V}}$O_2max_ to performance.

### Methodological considerations for future studies

Research on exercise intensity normalisation predominantly falls into three categories: a) those that demonstrate the variability in work rate targets based on percentages of a maximal benchmark (e.g. 70%$\dot{\text{V}}$O_2max_, 60%$\dot{\text{W}}$_max_) in relation to the intensity domains of exercise (Katch et al. 1978; Iannetta et al. 2020); b) those that assess inter-individual variability in performance, physiological, and/or perceptual responses to acute bouts of exercise (McLellan and Skinner 1985; Lansley et al. 2011; Scharhag-Rosenberger et al. 2010; Vollaard et al. 2009; Meyler et al. 2023); and c) those that compare inter-individual variability in training adaptations between groups differing in how exercise intensity is normalised (McLellan and Skinner 1981; Preobrazenski et al. 2019; Weatherwax et al. 2019). The rationale linking these rather distinct experimental designs is that a large inter-individual variability in acute exercise responses is likely to manifest as a large variability in adaptive responses to a training programme (Mann et al. 2014; Meyler et al. 2021). This theory draws upon molecular biology evidence that chronic adaptations to training originate from the cumulative effects of transient homeostatic perturbations associated with each exercise session (Perry et al. 2010; Egan and Zierath 2013). The findings of the present study and others (McLellan and Skinner 1981; Preobrazenski et al. 2019) suggest that future investigations should look at acute exercise responses to different methods of exercise intensity normalisation rather than adaptive responses, thus avoiding waste of time and resources.

### Limitations

One relevant characteristic of our study is that the total training load was not only dependent on laboratory-based training sessions, unlike comparable investigations (Weatherwax et al. 2019; McLellan and Skinner 1981; Preobrazenski et al. 2019). While the experimental design (with a control phase prior to the intervention) and analytical procedures permitted that this source of variability was accounted for, we cannot discard the possibility that participants changed their habitual training routine after the start of the intervention, affecting the magnitude of inter-individual variability in adaptive responses. Despite our efforts to persuade participants of the importance of training load documentation, very few did so with sufficient detail to provide insights in this respect.

## Conclusions

In summary, this study suggests that $\dot{\text{W}}$_max-SP_ may be used to normalise the intensity of interval training performed at predefined work rates. This approach prevents premature exhaustion, although without necessarily minimising inter-individual variability in RPE and heart rate. The inter-individual variability in adaptive responses to training, while only detected for $\dot{\text{V}}$O_2max_ and $\dot{\text{W}}$_max-SP_ amongst six variables, was similar in magnitude between groups differing (only) in how exercise intensity was normalised (i.e. 100%$\dot{\text{W}}$_max-SP_ vs. 80%$\dot{\text{W}}$_max_). Furthermore, no between-group differences in the magnitude of average responses to training were observed across all variables. These results underline the complexity of the relationship between acute training dose and chronic adaptations. From a methodological point of view, true inter-individual variability in adaptive responses cannot always be identified when intra-individual variability is accounted for.

### Supplementary Information

Below is the link to the electronic supplementary material.

Supplementary file1 (PDF 271 KB)

## Data Availability

The datasets generated during the current study are available from the corresponding author on reasonable request.

## Abbreviations

%VT_1_: Fraction of an individual’s first ventilatory threshold
%$\dot{\text{W}}$_max_: Group in which training intensity was prescribed relative to the maximal work rate achieved in an incremental test
%$\dot{\text{W}}$_max-SP_: Group in which training intensity was prescribed relative to the maximal sustainable work rate in a self-paced interval training session
[La^−^]: Blood lactate concentration
4 mmol·L^−^^1^_PO_: Power output associated with 4 mmol·L^−^^1^ blood lactate concentration
ANT +: Proprietary ultra-low power wireless standard for sensor data collection
CV: Coefficient of variation
GE: Gross efficiency
ICC: Intraclass correlation coefficient
N/A: Not applicable
P: Probability of obtaining the observed results assuming that the null hypothesis is true
Q1: Twenty-fifth percentile
Q3: Seventy-fifth percentile
r: Correlation coefficient
R^2^: Coefficient of determination
RPE: Rating of perceived exertion
SD: Standard deviation
SE: Standard error
$\dot{\text{V}}$O_2max_: Maximal oxygen uptake
VT_1_: First ventilatory threshold
$\dot{\text{W}}$_max_: Maximal work rate of an incremental test to exhaustion
$\dot{\text{W}}$_max-SP_: Maximal sustainable work rate of a self-paced interval training session
Δ$\mathop {\text{V}}\limits^{.}$O_2max_: Individual estimates for weekly changes in maximal oxygen uptake
Δ$\dot{\text{W}}$_max-SP_: Individual estimates for weekly changes in the maximal sustainable work rate in a self-paced interval training session

## Code availability

R code is available from the corresponding author on reasonable request.

## References

[CR1] Astorino TA, deRevere J, Anderson T, Kellogg E, Holstrom P, Ring S, Ghaseb N (2018). Change in V̇O2max and time trial performance in response to high-intensity interval training prescribed using ventilatory threshold. Eur J Appl Physiol.

[CR2] Atkinson G, Williamson P, Batterham AM (2019). Issues in the determination of ‘responders’ and ‘non-responders’ in physiological research. Exp Physiol.

[CR3] Björklund G, Pettersson S, Schagatay E (2007). Performance predicting factors in prolonged exhausting exercise of varying intensity. Eur J Appl Physiol.

[CR4] Black MI, Jones AM, Bailey SJ, Vanhatalo A (2015). Self-pacing increases critical power and improves performance during severe-intensity exercise. Appl Physiol Nutr Metab.

[CR5] Bonafiglia JT, Ross R, Gurd BJ (2019). The application of repeated testing and monoexponential regressions to classify individual cardiorespiratory fitness responses to exercise training. Eur J Appl Physiol.

[CR6] Borg GA (1982). Psychophysical bases of perceived exertion. Med Sci Sports Exerc.

[CR7] Bossi AH, Mesquida C, Passfield L, Rønnestad BR, Hopker JG (2020). Optimizing interval training through power-output variation within the work intervals. Int J Sports Physiol Perform.

[CR8] Bouchard C, An P, Rice T, Skinner JS, Wilmore JH, Gagnon J, Perusse L, Leon AS, Rao DC (1999). Familial aggregation of V̇O2max response to exercise training: results from the HERITAGE family study. J Appl Physiol.

[CR9] Bouchard C, Sarzynski MA, Rice TK, Kraus WE, Church TS, Sung YJ, Rao DC, Rankinen T (2011). Genomic predictors of the maximal O2 uptake response to standardized exercise training programs. J Appl Physiol.

[CR10] Brosnan MJ, Martin DT, Hahn AG, Gore CJ, Hawley JA (2000). Impaired interval exercise responses in elite female cyclists at moderate simulated altitude. J Appl Physiol.

[CR11] Brown VA (2021). An introduction to linear mixed-effects modeling in R. Adv Methods Pract Psychol Sci.

[CR12] Coakley SL, Passfield L (2018). Individualised training at different intensities, in untrained participants, results in similar physiological and performance benefits. J Sports Sci.

[CR13] Daniels JT, Yarbrough RA, Foster C (1978). Changes in V̇O2max and running performance with training. Eur J Appl Physiol Occup Physiol.

[CR14] Del Giudice M, Bonafiglia JT, Islam H, Preobrazenski N, Amato A, Gurd BJ (2020). Investigating the reproducibility of maximal oxygen uptake responses to high-intensity interval training. J Sci Med Sport.

[CR15] Egan B, Zierath JR (2013). Exercise metabolism and the molecular regulation of skeletal muscle adaptation. Cell Metab.

[CR16] Ferguson C, Wilson J, Birch KM, Kemi OJ (2013). Application of the speed-duration relationship to normalize the intensity of high-intensity interval training. PLoS ONE.

[CR17] Gaskill SE, Walker AJ, Serfass RA, Bouchard C, Gagnon J, Rao DC, Skinner JS, Wilmore JH, Leon AS (2001). Changes in ventilatory threshold with exercise training in a sedentary population: the HERITAGE family study. Int J Sports Med.

[CR18] Hecksteden A, Faude O, Meyer T, Donath L (2018). How to construct, conduct and analyze an exercise training study?. Front Physiol.

[CR19] Hecksteden A, Pitsch W, Rosenberger F, Meyer T (2018). Repeated testing for the assessment of individual response to exercise training. J Appl Physiol.

[CR20] Hickson RC, Hagberg JM, Ehsani AA, Holloszy JO (1981). Time course of the adaptive responses of aerobic power and heart rate to training. Med Sci Sports Exerc.

[CR21] Hopker J, Passfield L, Coleman D, Jobson S, Edwards L, Carter H (2009). The effects of training on gross efficiency in cycling: a review. Int J Sports Med.

[CR22] Iannetta D, Inglis EC, Mattu AT, Fontana FY, Pogliaghi S, Keir DA, Murias JM (2020). A critical evaluation of current methods for exercise prescription in women and men. Med Sci Sports Exerc.

[CR23] Jamnick NA, Pettitt RW, Granata C, Pyne DB, Bishop DJ (2020). An examination and critique of current methods to determine exercise intensity. Sports Med.

[CR24] Jones AM, Vanhatalo A (2017). The 'critical power' concept: applications to sports performance with a focus on intermittent high-intensity exercise. Sports Med.

[CR25] Joyner MJ, Coyle EF (2008). Endurance exercise performance: the physiology of champions. J Physiol.

[CR26] Joyner MJ, Lundby C (2018). Concepts about V̇ O2max and trainability are context dependent. Exerc Sport Sci Rev.

[CR27] Katch V, Weltman A, Sady S, Freedson P (1978). Validity of the relative percent concept for equating training intensity. Eur J Appl Physiol Occup Physiol.

[CR28] Lansley KE, Dimenna FJ, Bailey SJ, Jones AM (2011). A ‘new’ method to normalise exercise intensity. Int J Sports Med.

[CR29] Mann T, Lamberts RP, Lambert MI (2013). Methods of prescribing relative exercise intensity: physiological and practical considerations. Sports Med.

[CR30] Mann TN, Lamberts RP, Lambert MI (2014). High responders and low responders: factors associated with individual variation in response to standardized training. Sports Med.

[CR31] Marini CF, Sisti D, Leon AS, Skinner JS, Sarzynski MA, Bouchard C, Rocchi MBL, Piccoli G, Stocchi V, Federici A, Lucertini F (2021). HRR and V̇ O2R fractions are not equivalent: is it time to rethink aerobic exercise prescription methods?. Med Sci Sports Exerc.

[CR32] McLellan TM, Skinner JS (1981). The use of the aerobic threshold as a basis for training. Can J Appl Sport Sci.

[CR33] McLellan TM, Skinner JS (1985). Submaximal endurance performance related to the ventilation thresholds. Can J Appl Sport Sci.

[CR34] Meyler S, Bottoms L, Muniz-Pumares D (2021). Biological and methodological factors affecting V̇O2max response variability to endurance training and the influence of exercise intensity prescription. Exp Physiol.

[CR35] Meyler S, Bottoms L, Wellsted D, Muniz-Pumares D (2023). Variability in exercise tolerance and physiological responses to exercise prescribed relative to physiological thresholds and to maximum oxygen uptake. Exp Physiol.

[CR36] Midgley AW, McNaughton LR, Carroll S (2007). Reproducibility of time at or near VO2max during intermittent treadmill running. Int J Sports Med.

[CR37] Montero D, Lundby C (2017). Refuting the myth of non-response to exercise training: ‘non-responders’ do respond to higher dose of training. J Physiol.

[CR38] Naumova EN, Must A, Laird NM (2001). Tutorial in biostatistics: evaluating the impact of ‘critical periods’ in longitudinal studies of growth using piecewise mixed effects models. Int J Epidemiol.

[CR39] Nicolò A, Bazzucchi I, Haxhi J, Felici F, Sacchetti M (2014). Comparing continuous and intermittent exercise: an “isoeffort” and “isotime” approach. PLoS ONE.

[CR40] Péronnet F, Massicotte D (1991). Table of nonprotein respiratory quotient: an update. Can J Sport Sci.

[CR41] Perry CG, Lally J, Holloway GP, Heigenhauser GJ, Bonen A, Spriet LL (2010). Repeated transient mRNA bursts precede increases in transcriptional and mitochondrial proteins during training in human skeletal muscle. J Physiol.

[CR42] Pinheiro JC, Bates DM (2020). Mixed-effects models in S and S-PLUS.

[CR43] Preobrazenski N, Bonafiglia JT, Nelms MW, Lu S, Robins L, LeBlanc C, Gurd BJ (2019). Does blood lactate predict the chronic adaptive response to training: a comparison of traditional and talk test prescription methods. Appl Physiol Nutr Metab.

[CR44] Reed JL, Pipe AL (2014). The talk test: a useful tool for prescribing and monitoring exercise intensity. Curr Opin Cardiol.

[CR45] Rønnestad BR, Hansen J, Nygaard H, Lundby C (2020). Superior performance improvements in elite cyclists following short-interval vs effort-matched long-interval training. Scand J Med Sci Sports.

[CR46] Rossiter HB (2011). Exercise: kinetic considerations for gas exchange. Compr Physiol.

[CR47] Scharhag-Rosenberger F, Meyer T, Gassler N, Faude O, Kindermann W (2010). Exercise at given percentages of VO2max: heterogeneous metabolic responses between individuals. J Sci Med Sport.

[CR48] Seiler S, Sylta Ø (2017). How does Interval-training prescription affect physiological and perceptual responses?. Int J Sports Physiol Perform.

[CR49] Seiler S, Jøranson K, Olesen BV, Hetlelid KJ (2013). Adaptations to aerobic interval training: interactive effects of exercise intensity and total work duration. Scand J Med Sci Sports.

[CR50] Shephard RJ, Rankinen T, Bouchard C (2004). Test-retest errors and the apparent heterogeneity of training response. Eur J Appl Physiol.

[CR51] Sjödin B, Jacobs I (1981). Onset of blood lactate accumulation and marathon running performance. Int J Sports Med.

[CR52] Thomas K, Stone M, St Clair Gibson A, Thompson K, Ansley L (2013). The effect of an even-pacing strategy on exercise tolerance in well-trained cyclists. Eur J Appl Physiol.

[CR53] Villerius V, Duc S, Grappe F (2008). Physiological and neuromuscular responses of competitive cyclists during a simulated self-paced interval training session. Int J Sports Med.

[CR54] Voisin S, Jacques M, Lucia A, Bishop DJ, Eynon N (2019). Statistical considerations for exercise protocols aimed at measuring trainability. Exerc Sport Sci Rev.

[CR55] Vollaard NB, Constantin-Teodosiu D, Fredriksson K, Rooyackers O, Jansson E, Greenhaff PL, Timmons JA, Sundberg CJ (2009). Systematic analysis of adaptations in aerobic capacity and submaximal energy metabolism provides a unique insight into determinants of human aerobic performance. J Appl Physiol.

[CR56] Weatherwax RM, Harris NK, Kilding AE, Dalleck LC (2019). Incidence of V̇O2max responders to personalized versus standardized exercise prescription. Med Sci Sports Exerc.

[CR57] Williamson PJ, Atkinson G, Batterham AM (2017). Inter-individual responses of maximal oxygen uptake to exercise training: a critical review. Sports Med.

