# Supplementary material for: Modelling inter-individual variability in acute and adaptive responses to interval training: insights into exercise intensity normalisation
Source: Eur J Appl Physiol. 2023 Nov 15;124(4):1201–16. doi: 10.1007/s00421-023-05340-y (PMC10954971; doi:10.1007/s00421-023-05340-y)
Supplement: Supplementary file 1 — Supplementary file1 (PDF 271 KB) [file 421_2023_5340_MOESM1_ESM.pdf]

Day-to-day reliability estimates for each testing week.

Measure	$\dot{V}O_{2max}$ (L·min^{-1})				$\dot{W}_{max}$ (W)				4 mmol·L^{-1}$_{PO}$ (W)				GE (%)			
Week	0	7	11	15	0	7	11	15	0	7	11	15	0	7	11	15
Typical Error	0.115	0.145	0.115	0.130	9	5	10	4	7	7	8	6	0.7	0.7	0.6	0.8
CV (%)	2.9	3.7	2.7	3.1	2.4	1.2	2.9	1.1	3.1	2.7	3.7	2.7	3.9	3.5	3.1	4.0
ICC	0.96	0.93	0.97	0.96	0.95	0.99	0.95	0.99	0.97	0.98	0.96	0.98	0.87	0.83	0.81	0.83

CV, coefficient of variation; ICC, intraclass correlation coefficient; $\dot{V}O_{2max}$, maximal oxygen uptake; $\dot{W}_{max}$, maximal work rate in an incremental test; 4 mmol·L^{-1}$_{PO}$, power output associated with 4 mmol·L^{-1} blood lactate concentration; GE, gross efficiency. A freely available spreadsheet was used to calculate reliability estimates (Hopkins 2015 Sportscience 19:36-42).

Linear mixed model estimates for acute training responses.

	Random Effects (SD)		
	RPE	Heart Rate (beats·min^{-1})	Cadence (rev·min^{-1})
Intercept	0.8	9	6
Residual	0.8	3	3

	Fixed Effects								
	RPE			Heart Rate (beats·min^{-1})			Cadence (rev·min^{-1})		
	Estimate	SE	P	Estimate	SE	P	Estimate	SE	P
Intercept	15.4	0.3	< 0.001	173	3	< 0.001	96	2	< 0.001
$\%\dot{W}_{max\text{-}SP}$	-0.5	0.4	0.204	-7	4	0.089	0	3	0.988
Training Session 2	-0.2	0.1	0.061	-1	1	0.108	1	1	0.146
Training Session 3	-0.5	0.1	< 0.001	-1	1	0.009	1	1	0.054
Training Session 4	-0.4	0.1	0.001	0	1	0.989	2	1	0.004
Training Session 5	-0.5	0.1	< 0.001	-2	1	< 0.001	0	1	0.399
Training Session 6	-0.7	0.1	< 0.001	-1	1	0.075	3	1	< 0.001
Training Session 7	-0.3	0.1	0.013	1	1	0.056	2	1	0.001
Training Session 8	-0.3	0.1	0.033	1	1	0.013	2	1	< 0.001
Training Session 9	-0.5	0.1	< 0.001	1	1	0.131	1	1	0.023
Training Session 10	-0.6	0.1	< 0.001	1	1	0.108	2	1	< 0.001
Training Session 11	-0.5	0.1	< 0.001	1	1	0.044	2	1	0.006
Training Session 12	-0.6	0.1	< 0.001	2	1	0.002	2	1	< 0.001
Work Interval 2	1.2	0.1	< 0.001	4	0	< 0.001	0	0	0.376
Work Interval 3	2.4	0.1	< 0.001	6	0	< 0.001	0	0	0.232
Work Interval 4	3.3	0.1	< 0.001	8	0	< 0.001	-1	0	0.002
Work Interval 5	4.1	0.1	< 0.001	9	0	< 0.001	-1	0	< 0.001
Work Interval 6	4.6	0.1	< 0.001	9	0	< 0.001	-1	0	0.009

Formula: dependent variable = intercept + group estimate + training session estimate + work interval estimate. For $\%\dot{W}_{max}$, training session 1, and work interval 1, consider an estimate of 0. $\%\dot{W}_{max}$, group in which training intensity was prescribed relative to the maximal work rate achieved in an incremental test; $\%\dot{W}_{max\text{-}SP}$, group in which training intensity was prescribed relative to the maximal sustainable work rate in a self-paced interval training session; RPE, ratings of perceived exertion.

Linear mixed model estimates for maximal oxygen uptake ($L \cdot min^{-1}$).

Random Effect	Intercept	Intervention Week	Residual
SD	0.502	0.027	0.139

		Model Coefficients			Model Predictions							
					Control				Intervention		Gain	
Participant	Group	Intercept	Control Week	Intervention Week	Week 0	Week 7	Week 11	Week 15	Week 11	Week 15	Week 11	Week 15
1		3.765	0.009	0.020	3.765	3.826	3.861	3.896	3.940	4.054	0.079	0.158
3		4.312	0.009	0.045	4.312	4.373	4.408	4.443	4.589	4.805	0.181	0.361
5		3.551	0.009	-0.005	3.551	3.612	3.647	3.682	3.629	3.645	-0.018	-0.037
7		4.869	0.009	0.042	4.869	4.930	4.965	5.000	5.132	5.333	0.166	0.333
9		4.406	0.009	0.012	4.406	4.467	4.502	4.537	4.550	4.633	0.048	0.096
11	%$\dot{W}_{max}$	3.574	0.009	-0.012	3.574	3.636	3.670	3.705	3.624	3.613	-0.046	-0.092
13		3.118	0.009	-0.009	3.118	3.179	3.214	3.249	3.177	3.175	-0.037	-0.074
15		3.854	0.009	0.015	3.854	3.915	3.950	3.985	4.010	4.106	0.060	0.121
17		4.606	0.009	0.062	4.606	4.667	4.702	4.737	4.949	5.230	0.246	0.493
19		4.271	0.009	0.024	4.271	4.332	4.367	4.402	4.463	4.594	0.096	0.192
Mean		4.033		0.019					4.206	4.319	0.078	0.155
SD		0.546		0.024					0.631	0.718	0.098	0.196
2		4.106	0.009	0.045	4.106	4.168	4.203	4.237	4.381	4.594	0.178	0.356
4		4.556	0.009	0.027	4.556	4.617	4.652	4.687	4.762	4.906	0.110	0.219
6		3.672	0.009	0.006	3.672	3.733	3.768	3.803	3.794	3.855	0.026	0.052
8		3.792	0.009	-0.007	3.792	3.853	3.888	3.923	3.861	3.869	-0.027	-0.053
10	%$\dot{W}_{max-SP}$	4.550	0.009	-0.001	4.550	4.611	4.646	4.681	4.644	4.676	-0.002	-0.005
12		4.475	0.009	0.019	4.475	4.536	4.571	4.606	4.645	4.754	0.074	0.148
14		3.667	0.009	-0.011	3.667	3.728	3.763	3.798	3.721	3.714	-0.042	-0.085
16		4.380	0.009	0.030	4.380	4.441	4.476	4.511	4.597	4.753	0.121	0.243
18		4.886	0.009	0.042	4.886	4.947	4.982	5.017	5.150	5.353	0.168	0.336
Mean		4.231		0.017					4.395	4.497	0.067	0.135
SD		0.441		0.021					0.496	0.558	0.083	0.165

Formula: maximal oxygen uptake = intercept + control week coefficient · x + intervention week coefficient · x. For control week, x = 0 to 15; for intervention week, x = 0 to 8 (where intervention week 1 corresponds to control week 8). %$\dot{W}_{max}$, group in which training intensity was prescribed relative to the maximal work rate achieved in an incremental test; %$\dot{W}_{max-SP}$, group in which training intensity was prescribed relative to the maximal sustainable work rate in a self-paced interval training session.

Linear mixed model estimates for the maximal sustainable work rate in a 6 × 4 min self-paced interval training session (W).

Random Effect	Intercept	Intervention Week	Residual
SD	39	1.451	5.440

		Model Coefficients			Model Predictions							
					Control				Intervention		Gain	
Participant	Group	Intercept	Control Week	Intervention Week	Week 0	Week 7	Week 11	Week 15	Week 11	Week 15	Week 11	Week 15
1		272	0.578	-1.915	272	276	278	281	271	265	-8	-15
3		298	0.578	2.369	298	302	305	307	314	326	9	19
5		240	0.578	-0.723	240	244	246	249	243	243	-3	-6
7		332	0.578	1.521	332	336	339	341	345	353	6	12
9		292	0.578	0.389	292	296	298	300	299	303	2	3
11	%$\dot{W}_{max}$	262	0.578	-0.227	262	266	268	270	267	268	-1	-2
13		220	0.578	1.190	220	224	226	229	231	238	5	10
15		231	0.578	2.205	231	235	237	239	246	257	9	18
17		325	0.578	1.814	325	329	331	333	338	348	7	15
19		297	0.578	0.406	297	301	303	305	305	309	2	3
Mean		277		0.703					286	291	3	6
SD		39		1.379					40	42	6	11
2		329	0.578	-1.733	329	333	335	338	328	324	-7	-14
4		346	0.578	-0.926	346	350	353	355	349	347	-4	-7
6		240	0.578	0.964	240	244	247	249	251	257	4	8
8		260	0.578	-0.020	260	264	266	269	266	268	0	0
10	%$\dot{W}_{max-SP}$	308	0.578	0.628	308	312	315	317	317	322	3	5
12		301	0.578	0.044	301	305	307	309	307	310	0	0
14		249	0.578	-0.415	249	253	256	258	254	255	-2	-3
16		265	0.578	2.132	265	269	271	273	280	290	9	17
18		333	0.578	0.694	333	337	339	342	342	347	3	6
Mean		292		0.152					299	302	1	1
SD		40		1.128					38	36	5	9

Formula: maximal sustainable work rate = intercept + control week coefficient · *x* + intervention week coefficient · *x*. For control week, *x* = 0 to 15; for intervention week, *x* = 0 to 8 (where intervention week 1 corresponds to control week 8). %$\dot{W}_{max}$, group in which training intensity was prescribed relative to the maximal work rate achieved in an incremental test; %$\dot{W}_{max-SP}$, group in which training intensity was prescribed relative to the maximal sustainable work rate in a self-paced interval training session.
